# Structural Brain Changes after Traditional and Robot-Assisted Multi-Domain Cognitive Training in Community-Dwelling Healthy Elderly

**DOI:** 10.1371/journal.pone.0123251

**Published:** 2015-04-21

**Authors:** Geon Ha Kim, Seun Jeon, Kiho Im, Hunki Kwon, Byung Hwa Lee, Ga Young Kim, Hana Jeong, Noh Eul Han, Sang Won Seo, Hanna Cho, Young Noh, Sang Eon Park, Hojeong Kim, Jung Won Hwang, Cindy W. Yoon, Hee Jin Kim, Byoung Seok Ye, Ju Hee Chin, Jung-Hyun Kim, Mee Kyung Suh, Jong Min Lee, Sung Tae Kim, Mun-Taek Choi, Mun Sang Kim, Kenneth M Heilman, Jee Hyang Jeong, Duk L. Na

**Affiliations:** 1 Department of Neurology, Ewha Womans University Mokdong Hospital, Ewha Womans University School of Medicine, Seoul, Korea; 2 Department of Neurology, Samsung Medical Center, Sungkyunkwan University School of Medicine, Seoul, Korea; 3 Department of Biomedical Engineering, Hanyang University, Seoul, Korea; 4 Division of Newborn Medicine, Boston Children′s Hospital, Harvard Medical School, Boston, Massachustetts, United States of America; 5 Department of Neurology, Asan Medical Center, University of Ulsan College of Medicine, Seoul, Korea; 6 Department of Neurology, Gangnam Severance Hospital, Yonsei University College of Medicine, Seoul, Korea; 7 Department of Neurology, Gachon University Gil Medical Center, Incheon, Korea; 8 Samsung Advanced Institute for Health Sciences and Technology, Sungkyunkwan University, Korea; 9 Department of Neurology, Inha University Hospital, Inha University School of Medicine, Incheon, Korea; 10 Department of Neurology, Yonsei University College of Medicine, Seoul, Korea; 11 Department of Radiology, Samsung Medical Center, Sungkyunkwan University School of Medicine, Seoul, Korea; 12 School of Mechanical Engineering, Sungkyunkwan University, Seoul, Korea; 13 Center for Intelligent Robotics at Korea Institute Science and Technology, Seoul, Korea; 14 Department of Neurology, University of Florida College of Medicine, and the Veterans Affairs Medical Center, Gainesville, Florida, United States of America; MRC Institute of Hearing Research, UNITED KINGDOM

## Abstract

The purpose of this study was to investigate if multi-domain cognitive training, especially robot-assisted training, alters cortical thickness in the brains of elderly participants. A controlled trial was conducted with 85 volunteers without cognitive impairment who were 60 years old or older. Participants were first randomized into two groups. One group consisted of 48 participants who would receive cognitive training and 37 who would not receive training. The cognitive training group was randomly divided into two groups, 24 who received traditional cognitive training and 24 who received robot-assisted cognitive training. The training for both groups consisted of daily 90-min-session, five days a week for a total of 12 weeks. The primary outcome was the changes in cortical thickness. When compared to the control group, both groups who underwent cognitive training demonstrated attenuation of age related cortical thinning in the frontotemporal association cortices. When the robot and the traditional interventions were directly compared, the robot group showed less cortical thinning in the anterior cingulate cortices. Our results suggest that cognitive training can mitigate age-associated structural brain changes in the elderly.

**Trial Registration:**

ClnicalTrials.gov NCT01596205

## Introduction

The growing population of elderly coupled with the absence of definitive treatments for age-related cognitive decline has prompted efforts to find strategies that can potentially reduce this decline. Several studies have reported that cognitive training may help maintain or improve cognition in the elderly [1] and could therefore be an effective means by which late life cognitive impairment can be abated [2].

In addition to cognitive decline, studies have revealed that with aging there is thinning of the cerebral cortex [3] and studies have also investigated structural brain plasticity after cognitive training in the elderly [4, 5], demonstrating that cognitive training increases regional gray matter volume. However, in most studies, cognitive training only targeted a single cognitive domain, such as memory [4, 5]. Single domain cognitive intervention may have theoretical importance since it may allow researchers to investigate direct training-related effects [6] but multi-domain training could potentially have more practical advantages than single-domain training because multiple cognitive functions are required for humans to live in the world [6, 7]. In addition, individuals receiving multi-domain cognitive training may benefit more in terms of maintenance of training effects than those receiving single-domain training perhaps because of the transfer effect [7, 8]. The transfer effect refers to improvement on an untrained cognitive domain after receiving a specific cognitive training [9]. It is therefore possible that multi-domain cognitive training would elicit more synergistic transfer effects across domains than single-domain cognitive training since it targets multiple cognitive functions [10, 11]. Despite these findings, there has been only one study that evaluated the structural brain changes following multi-domain cognitive training, and this study examined a relatively small number of participants [12].

With the advent of robotics, service robots that can interact with humans have attracted both industry and academic interest [13]. In particular, robots to assist the elderly may be important given the rapid increase in the aging population and the exorbitant healthcare costs associated with caring for older individuals with cognitive decline. Furthermore, traditional cognitive training with paper and pencil usually needs experienced instructors [9], but those qualified instructors may be unavailable in some chronic care facilities or community centers. For this reason, we have developed a total of 17 robot-assisted cognitive training programs for the elderly.

The goal of this study was to test the hypothesis that multi-domain cognitive training would delay age-associated cortical thinning and structural network alterations in the brains of the elderly. In addition, we also wanted to investigate if robot-assisted cognitive training would result in greater effects than traditional cognitive training since exposure to new technology is likely to be more challenging for the elderly than familiar technologies and novelty may increase brain activity [10, 11]. To test these hypotheses, we compared changes in cortical thickness and structural connectivity of cognitive training groups to a control group without cognitive training. Then, we compared the effects of our newly developed, robot-assisted, multi-domain cognitive training programs on brain structures to traditional human-assisted cognitive training programs.

## Methods

### Study design

This randomized controlled study on the effects of 12 week- cognitive training on changes of cortical thickness in healthy elderly participants was conducted between June 2011 and May 2012 at Samsung Medical Center in Seoul, Republic of Korea. This trial was registered at “ClnicalTrials.gov” as NCT01596205.

### Participants

**Fig 1:**
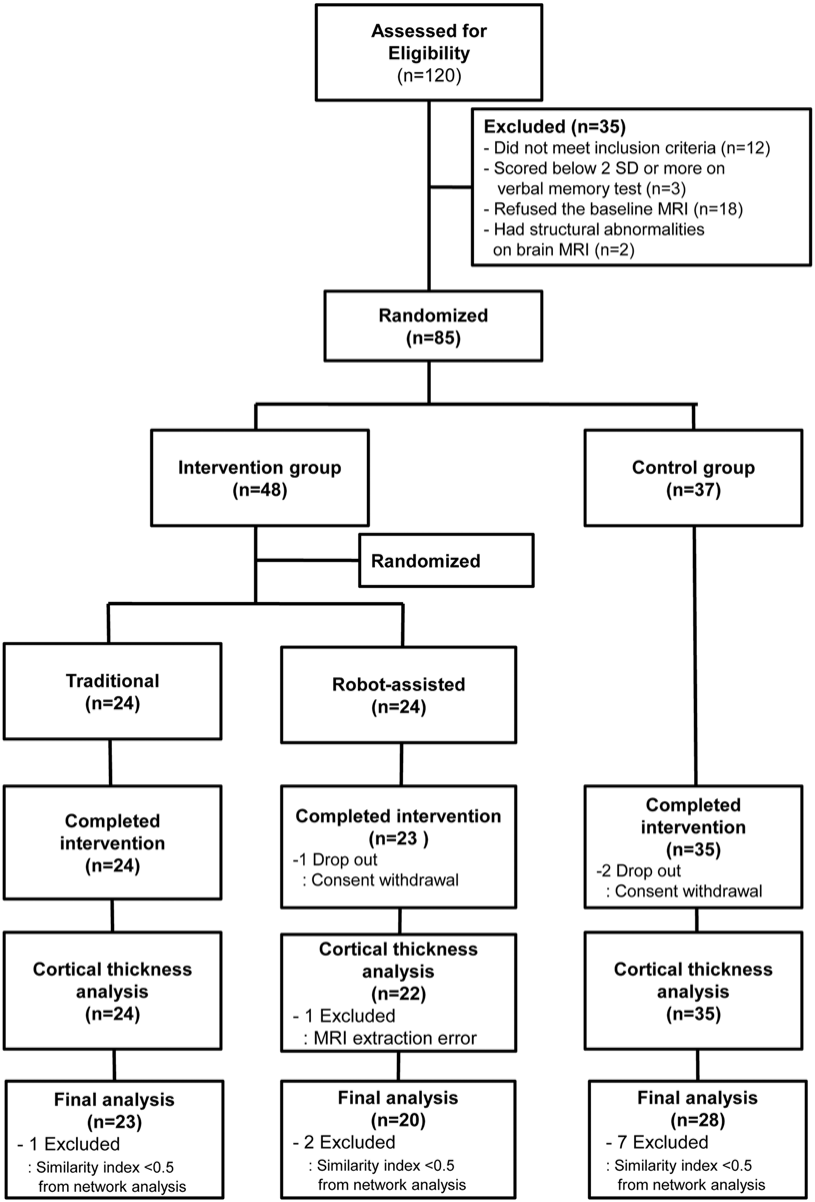
Flow of participants in this study. The similarity index was defined as follows:similarity index = 2 * nnz(A and B)/(nnz(A) + nnz(B)) where A and B are the baseline and post-intervention connectivity from binary matrices, respectively and nnz refers to the number of non-zero elements in a matrix. If the two binary matrices were the same, the similarity index was assigned a value of 1. We excluded subjects with a similarity index lower than 0.5 in our statistical analyses to reduce the artifactual effects related to the different times of scanning.

Community-dwelling volunteers aged 60 years or older were recruited from the Gangnam Center for Dementia, one of the public facilities for dementia prevention in Seoul. Normally this center, as a service to the community, screens about 6000 elderly annually using the Mini-Mental State Examination (MMSE) for early detection of dementia. Among a total of 2877 individuals who were screened from March to August in 2011 at Gangnam Center for Dementia, 534 who had Korean version of MMSE scores (K-MMSE) [14] ≥ 26 and more than 6 years of education were initially contacted for participation. Among them, 120 agreed to participate in this study. A neurologist evaluated eligibility using the following inclusion criteria: literacy, no known history of dementia or significant cognitive impairment, no visual or hearing impairment severe enough to interfere with cognitive testing/cognitive training, no history of major neurological or psychiatric illnesses, no history of medication that could affect cognitive function and no major medical problems. Of those 120 participants,12 individuals were excluded for the following reasons: five had a history of stroke, four were taking antidepressants and three had thyroid dysfunction. To minimize the influence of subclinical degenerative conditions, three participants were excluded who scored two standard deviations (SD) or more below population norm on the tests of immediate learning and/or delayed recall scores of Seoul Verbal Learning Tests [15]. Another 18 were excluded because they refused MRI. Two persons were additionally excluded after taking MRI because one had pituitary adenoma while the other had an old posterior cerebral artery territorial infarction on MRI. Therefore, the final sample consisted of 85 participants (Fig 1). Written informed consent was obtained from all participants and the study protocol was approved by the institutional review board of Samsung Medical Center (IRB 2011-04-080).

### Randomization

After baseline assessments, 85 participants were randomly assigned into the two groups: an intervention group of 48 patients and non-intervention group of 37 patients using simple random sampling. Twenty-four patients from the intervention group were randomly selected and assigned to a robot-assisted intervention group (robot group), while the others were assigned to a traditional intervention group (traditional group) using SPSS software (Fig 1). The randomization process was performed by a researcher who was blinded to intervention. The intervention groups did not differ in terms of age, sex, years of education, baseline neuropsychological test results, physical activity, and cardiovascular risk factors, or number of carriers of the *APOE* ε4 allele compared to the control group (Table 1).

**Table 1:** Demographic and clinical characteristics of the participants.

	Intervention group (n = 48)			Control (n = 37)	*P* value (Intervention Vs. Control)
	Traditional (n = 24)	Robot (n = 24)	*P* value		
**Gender (Male: Female)**	6:18	10:14	0.359	9:28	0.473
**Age (years)**	67.7 ± 5.4	68.0 ± 6.1	0.823	66.9 ± 4.0	0.353
**Education (years)**	14.0 ± 3.3	13.2 ± 3.9	0.466	13.2 ± 3.7	0.619
**K-MMSE**	29.1 ± 0.9	28.9 ± 1.5	0.502	29.0 ± 1.3	0.924
**ADAS-Cog**	7.5 ± 3.5	6.8 ± 3.8	0.503	6.3 ± 3.6	0.310
**Risk factors, n (%)**					
** Diabetes Mellitus**	1 (4.2)	2 (8.7)	0.609	1 (2.7)	0.635
** Hypertension**	7 (29.2)	6 (25.0)	1.000	12 (32.4)	0.806
** Hyperlipidemia**	4 (16.7)	6 (25.0)	0.527	8 (21.6)	0.673
***APOE* ε4 allele, n (%)** ^**a**^			0.698		0.408
** 0 (-/-)**	18 (85.7)	17 (77.3)		27 (84.4)	
** 1(ε4 /-)**	3 (14.3)	5 (22.7)		4 (12.5)	
** 2 (ε4 / ε4)**	0 (0)	0 (0)		1 (3.1)	
**Physical activity (METs)**	3232.3 ± 2292.1	2714.7 ± 2545.5	0.769	3606.2 ± 3525.9	0.709
**Cortical thickness (mm)**	2.562 ± 0.125	2.561 ± 0.918	0.758	2.594 ± 0.106	0.174
**ICV(cm** ^**3**^ **)**	9944.0 ± 732.3	10217.1 ± 850.4	0.239	1007.9 ± 844.6	0.992

K-MMSE, Korean version of the Mini-Mental State Examination; ICV, intracerebral volume; MET, metabolic equivalent.

^a^ *APOE* was analyzed only in 75 patients because 10 patients refused the test.

### Interventions

Participants in both intervention groups were subdivided into three groups of eight participants. Each individual participant from the groups attended a total of 60 sessions, with each session being 90 minutes, five days a week for 12 weeks. Each intervention session consisted of an introduction (10 minutes), cognitive training (70 minutes), and final closing (10 minutes). The cognitive training portion of both intervention groups consisted of 2 training blocks that included five cognitive domains: memory, language, calculation, visuospatial function, and executive function. All participants in both intervention groups received 44 blocks for memory training, 14 for language, 12 for calculation, 16 for visuospatial function and 34 for executive function. All 85 participants received 10-hours of education about dementia prevention before undergoing baseline assessments. This education program was conducted for 2 hours per day from Monday to Friday. On Monday, the participants learned about human brain structures as well as cognitive functions, and on Tuesday, the prevention and treatments of dementia. On Wednesday, they were educated about the importance of cognitive training and they were provided with several examples of cognitive training they could perform at home. The healthy foods for brain as well as aerobic exercise and stretching [16, 17] that could be performed at home were discussed on Thursday and finally individual presentation of one’s thoughts about the overall program and laughter therapy [18] took place on Friday.

Two experienced psychometricians served as a facilitator or instructor in both traditional and robot-assisted cognitive training; therefore each psychometrician was in charge of three subgroups in the two intervention groups. The two psychometricians familiarized themselves with the manuals for cognitive training before study inception, and were instructed to adhere to these manuals, but were allowed to distribute the time flexibly among programs within the same cognitive domain. The details of traditional and robot interventions are provided in the S1 Methods. Table 2 contains a summary of the traditional and robot-assisted interventions.

**Table 2:** Comparisons of traditional and robot intervention.

	Traditional	Robot
**N of participants**	8 persons each group	8 persons each group
**N of psychometrician**	1	1
**Role of psychometrician**	Instructor	Assistant
**Methods of instruction**	Dictation (from screen) by psychometrician	Dictation (from screen) by robot
**Response methods**	Paper and pencil	Smart pad
**Scoring system**	By psychometrician	By robot
**Individual task scores**	Not stored	Stored
**Feedback**	Per group	Per individual and/or per group
**Contents**		
***Memory (44 blocks)***		
Verbal memory (Word learning)	10	10 (4 with motion)
Visual memory	8	8 (4 with motion)
Logical/story memory	8	8
Paired associate learning	10	10
Memory with song	8	8
***Language (14 blocks)***		
Word generation	6	6
Word comprehension	8	8
***Calculation (12 blocks)***		
Addition/subtraction/multiplication/division	12	12
***Visusospatial function (16 blocks)***		
Visuoconstruction	8	8 (4 with motion)
Topographical orientation	8	8 (4 with motion)
***Frontal executive function (34 blocks)***		
Working memory	14	14 (6 with motion)
Reasoning	8	8
Speed of processing/Attention	12	12 (8 with motion)

#### Traditional interventions

In traditional cognitive training, the psychometricians displayed questions on a screen positioned in the front of the intervention room. The participants attempted to answer these questions either verbally or in written form. The psychometricians then presented the correct answers on the screen.

#### Robot-assisted interventions

Silbot and Mero were the robots used in the robot-assisted cognitive training (S1 Fig). Except for three programs for which the robots detected and evaluated the participants’ motion, participants in the robot group responded to instructions using an individual smart pad (Galaxy tab 10.1, Samsung Electronics, Seoul, Korea). The same psychometricians instructed the participants in procedures used for cognitive training with these robots. The main roles of the psychometrician in the robot group were to select programs for participants as well as giving or repeating program instructions if necessary. Unlike the traditional training, robot-assisted training rewarded participants by giving participants individual feedback to each question immediately after entering the answers on their smart pads. Individual scores were saved at this point, and the winner of a day or the winner of a month was announced after completing each program. A total of 17 cognitive training programs were used in the robot-assisted cognitive training including five programs for memory, two for language, two for calculation, four for visuospatial function and four for executive function.

### Assessments and outcome measures

All participants underwent brain MRI and neuropsychological testing before and after training. All assessment and scans were conducted within 2 weeks before and after the intervention. The baseline physical activity of all participants was evaluated by the international physical activity questionnaire (IPAQ) [19]. The investigators who scored the neuropsychological tests and analyzed the MRI imaging were blinded to the participants’ intervention.

The primary outcome was the changes in cortical thickness as assessed by surface based morphometry (SBM), a method known to be one of the most sensitive and accurate means of measuring cortical thickness [20]. The secondary outcomes were changes in the structural brain network as assessed by a graph theoretical approach [21] and cognitive functions. Cognitive functions were assessed by Alzheimer’s Disease Assessment Scale—total score of the cognitive subscale (ADAS-cog) [22] and the seven subtests from the Cambridge Neuropsychological Test Automated Battery (CANTAB), which included the three visual memory tasks of Delayed Matching to Sample (DMS), Pattern Recognition Memory (PRM), and Paired Associates Learning (PAL), two executive function or working memory tests, namely Spatial Working Memory (SWM) and Stockings of Cambridge (SOC), and two attention tests, namely Reaction Time (RTI) and Rapid Visual Information Processing (RVIP). Detailed descriptions of the tests are available on Cambridge Cognition's website (http://www.cambridgecognition.com/academic/cantabsuite/tests) and S2 Methods.

#### MRI acquisition

All participants underwent brain MRI using identical imaging protocols on a 3.0 Tesla MRI scanner (Achieva, Philips 3.0T, Eindhoven, Netherlands). All participants were sent to Samsung Medical Center for MR imaging that was performed employing six different techniques (3D T1 TFE, FLAIR, T1 REF, T2, FFE and DTI) using the same imaging protocols and the same 3.0-Tesla MRI scanner (Achieva, Philips 3.0T, Eindhoven, Netherlands). For the analysis of cortical thickness, 3D T1 turbo field echo (TFE) MR images were acquired with the following imaging parameters: sagittal slice thickness, 1.0 mm; no gap; repetition time (TR), 9.9 ms; echo time (TE), 4.6 ms; flip angle, 8°; and a matrix size of 480x480 pixels. The second technique we used to evaluate the abnormal white matter hyperintensities was fluid attenuated inversion recovery (FLAIR) MR images, which were acquired with an axial slice thickness of 2 mm; no gap; TR of 11000.0 ms; TE of 125.0 ms; flip angle of 90°; and a matrix size of 512x512 pixels. The third technique was T1 reference (REF) MR imaging; images were acquired with an axial slice thickness of 4 mm; no gap; TR of 545 ms; TE of 10 ms; flip angle of 70°; and a matrix size of 512x512 pixels. To evaluate if there were abnormal signal intensities including lacunes, T2 MR images were acquired using an axial slice thickness of 5.0 mm; inter-slice thickness of 1.5 mm; TR of 3000.0 ms; TE of 80.0 ms; flip angle of 90°; and a matrix size of 512 x 512 pixels. To obtain the information about microbleeds, T2 fast field echo(FFE) images were obtained using the following parameters: axial slice thickness, 5.0 mm; inter-slice thickness, 2 mm; TR, 669 ms; TE 16 ms; flip angle, 18°, and a matrix size of 560x 560 pixels. All axial sections were obtained parallel to the anterior commissure-posterior commissure line. For the graph analysis of structural brain network, the diffusion tensor images (DTI) were acquired by diffusion-weighted single shot echo-planar imaging with the following parameters: TE, 60 ms; TR, 7,696 ms; flip angle, 90°; b-factor, 600 s/mm2; matrix dimensions, 128 x 128; 70 axial sections. With the baseline image without weighting, diffusion-weighted images were acquired from 45 different directions.

Data processing and analyses of MRI images were independently performed at the Computational Neuroimage Analysis Laboratory of Hanyang University, Seoul in Korea.

#### Cortical thickness measurements

Processing details are described in our previous studies [23, 24]. Briefly, native MRI images were registered into a standardized stereotaxic space using linear transformation [25]. The N3 algorithm was used to correct images for intensity nonuniformities resulting from inhomogeneities in the magnetic field [26]. The registered and corrected volumes were classified into white matter, grey matter, cerebrospinal fluid, and background using a 3D stereotaxic brain mask and the Intensity-Normalized Stereotaxic Environment for Classification of Tissues (INSECT) algorithm [27]. The surfaces of the inner and outer cortices were automatically extracted using the Constrained Laplacian-based Automated Segmentation with Proximities (CLASP) algorithm [28]. Cortical thickness was defined as the Euclidean distance between linked vertices of the inner and outer surfaces [29], because Lerch and Evans considered this method for the measurement of cortical thickness as the simplest and most precise among the several methods they evaluated [29].

#### Structural connectivity network construction and analyses

Nodes and edges are the two basic elements of a network. We defined nodes using an automated anatomical labelling (AAL) template [30] and edges as connections between two nodes. We measured nodal strength, global efficiency and the clustering coefficient using a similar procedure used in a previous study [31]. The procedure is described in S3 Methods.

Even though we used the same MRI scanner and the same imaging protocol, scanning subjects twice at two different time points may potentially result in differences of matricies between the pre- and post-intervention, which might not reflect real biological differences but rather imaging induced artifacts. To address this, we first converted weighted connectivity matrices into binary matrices and measured the similarity index between the baseline and the post-intervention for each subject. The similarity index was defined as follows: similarity index = 2 * nnz(A AND B)/(nnz(A) + nnz(B)) where A and B are the baseline and post-intervention connectivity from binary matrices, respectively and nnz refers to the number of non-zero elements in a matrix. If the two binary matrices were the same, the similarity index was assigned a value of 1. We excluded subjects with a similarity index lower than 0.5 in these statistical analyses to reduce the artificial effects related to the different times of scanning.

### Statistical analyses

We calculated our sample size based on a comparison of changes in cortical thickness from baseline to follow-up between the control and the intervention groups, as well as within the training groups comparing the traditional and the robot groups. For the normal age-associated cortical thinning, we referred to a longitudinal study from our group that compared changes of cortical thickness between normal control versus early or late-onset Alzheimer’s disease dementia, which showed that the mean change of cortical thinning in the normal control group (mean age 72.6 ± 2.7) was -0.021 ± 0.004 mm per year [32]. Based on this study, we regarded a cortical thinning of 0.005 mm per three months as significant in normal aging. A prior study from other group demonstrated that the control group (mean age 60.3± 9.1) showed an average cortical thinning by 0.005 mm for two months while the intervention group showed cortical *thickening* by 0.004 mm (the difference of between the two groups were 0.009 mm) [4]. Another study, however, showed that there were no significant changes of cortical thickness between the control and the intervention group in the elderly [5]. Taken these results together, we assumed that the difference in changes of cortical thickness between the control and the intervention group more than 0.004 mm is significant, which was about the average of those reported in the two previous studies.

Assuming the standard deviation (SD) of 0.004 mm for changes in cortical thickness from our previous study [32], and we expected changes in cortical thinning of -0.005 mm (0.6 months required for change of -0.001mm) for the control group, -0.004 mm (0.74 months required for change of -0.001 mm) for the traditional intervention group, and -0.001 mm (3 months required for change of -0.001 mm) for the robot-assisted intervention group. Therefore, the planned enrolment for the study was 24 per intervention group, and 37 for control group to account for 10% drop-out rate, which had 80% power under the 5% significance level.

Statistical analyses were implemented using SurfStat Matlab library (http://www.math.mcgill.ca/keith/surfstat; Math Works, Natick, MA) and PASW 18 (SPSS Inc., Chicago, IL, USA). For the baseline demographic data, student t-test was used to analyze continuous variables and the Chi-square test to analyze dichotomous variables. Global and topographical changes in mean cortical thickness and global network topology were compared between the groups using a general linear model (GLM) with age, gender and intracranial volume as covariates. Statistical significance was assessed by a permutation-based test with a threshold of uncorrected *P* < 0.005 [33, 34]. Changes in cognitive functions between the pre and post-intervention in each group were assessed by paired t-test while difference of changes in cognitive function between the groups were compared using a GLM with age, gender and years of education as covariates. Two-sided *P* < 0.05 was considered as statistical significance. Correlation between changes of cortical thickness and change of cognitive functions within each group was analyzed by GLM with age, gender and years of education as covariates, of which a permutation-based test was also used to assess the statistical significance with a threshold of uncorrected *P* < 0.001.

## Results

### Participants in the final analysis

The flow of participants through the study is depicted in Fig 1. One participant in the robot group and two in the control group withdrew consent. All 47 participants in the two intervention groups attended 51 or more (≥ 85%) of the 60 sessions and the attendance rate was not different between these two groups (56.0 ± 3.0 in the traditional vs. 55.9 ± 3.8 in the robot group, *P* = 0.864). Among the 82 participants who completed the intervention, one participant from the robot group was excluded since the extraction of cortical thickness had failed. For the secondary outcomes, one participant from the traditional group, two from the robot group, and seven from the control group, were excluded due to the similarity index lower than 0.5. Therefore, 71 participants were analyzed in the final analysis. Results of continuous variables were demonstrated as mean ± SD.

### Effects of cognitive training on cortical thickness

#### Global changes of cortical thickness

The mean changes of cortical thickness over 12 weeks in the control group were -0.056 ± 0.115 mm (95% CI, - 0.097 ~ - 0.016), which was significantly greater than those observed in the intervention group showing-0.011± 0.072 mm (95% CI, - 0.033 ~ 0.011) (delta for group differences = -0.046 ± 0.053, 95% CI, -0.150 ~ 0.058, *P* for group = 0.015). However, changes in the mean cortical thickness did not differ between the traditional (-0.013 ± 0.067 mm, 95% CI, - 0.042 ~ 0.016) and the robot group (- 0.008 ± 0.079 mm, 95% CI, -0.045 ~ 0.029), (delta for group differences = -0.006 ± 0.072 mm, 95% CI, - 0.147~ 0.135, *P* for group = 0.567).

As stated, we found an attenuation of cortical thinning in the intervention group compared to the control group. Therefore we investigated what would be the equivalent time of this attenuation in terms of brain-age. Given that the control group showed cortical thinning of 0.056 mm ± 0.115 mm for 3 months while the intervention group showed cortical thinning of 0.011 ± 0.072 mm, this difference was translated into 15.3 months, suggesting that the intervention group may take as long as 15.3 months to reach the same cortical thinning as in the control group.

#### Topographical changes of cortical thickness

**Fig 2:**
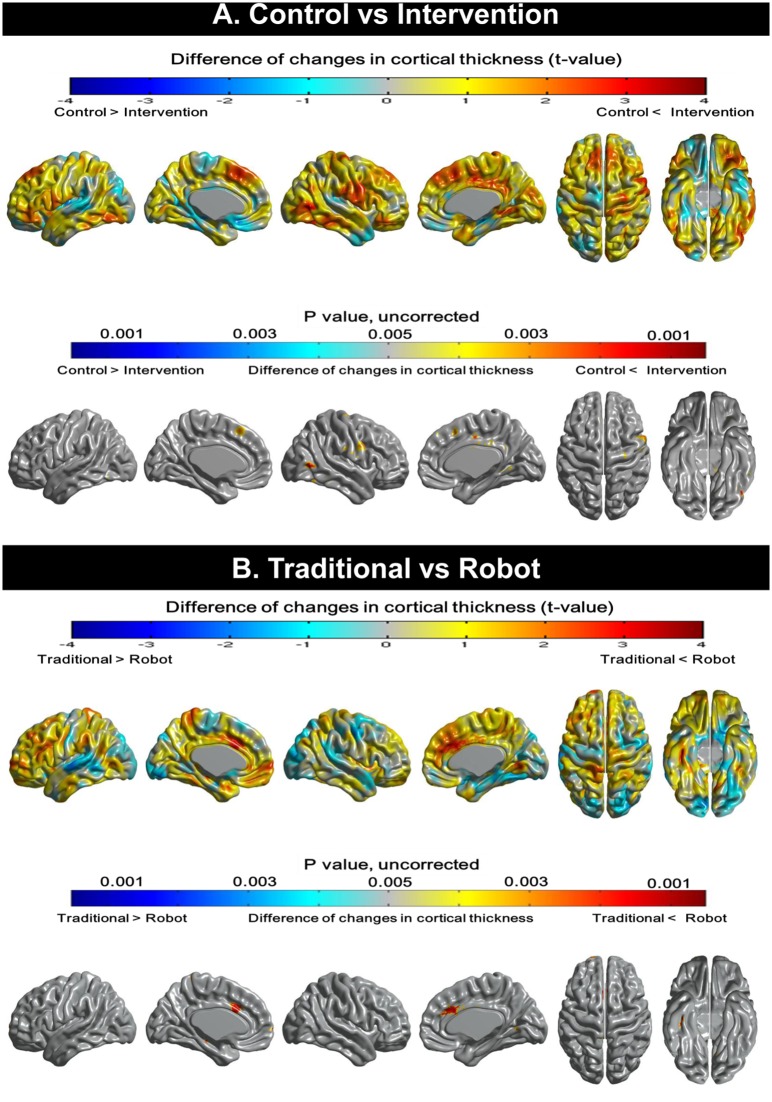
Topographical changes in cortical thickness. (A) Compared to the control group, the intervention group shows attenuated cortical thinning on heteromodal association cortices such as the bilateral medial prefrontal and right middle temporal gyrus. (B) When the traditional and robot groups were directly compared, significantly reduced cortical thinning on the bilateral anterior cingulate cortices and right inferior temporal cortex was evident in the robot group. No area demonstrated less cortical thinning in the traditional group than the robot group.

Fig 2A illustrates brain areas that showed significant changes in cortical thickness between the control and intervention group. Compared to the control group, the intervention group showed attenuated cortical thinning of heteromodal association cortices such as the bilateral medial prefrontal and right middle temporal gyrus (Fig 2A). When the robot and the traditional group were directly compared, the robot group had significantly reduced cortical thinning in the right and left anterior cingulate cortices (ACC) and small areas of right inferior temporal cortex compared to the traditional group whereas no area showed significantly reduced cortical thinning in the traditional group compared to the robot group (Fig 2B).

### Effects of cognitive training on structural network

#### Global changes of nodal strength

Global topological organization of white matter corticocortical networks such as nodal strength, global efficiency and clustering coefficient was decreased in the control group. There was no significant differences in the global topolgy of networks between traditional and robot intervention group although the rate of decrease was significantly less in the intervention groups (S1 Table).

#### Regional changes of nodal strength

**Fig 3:**
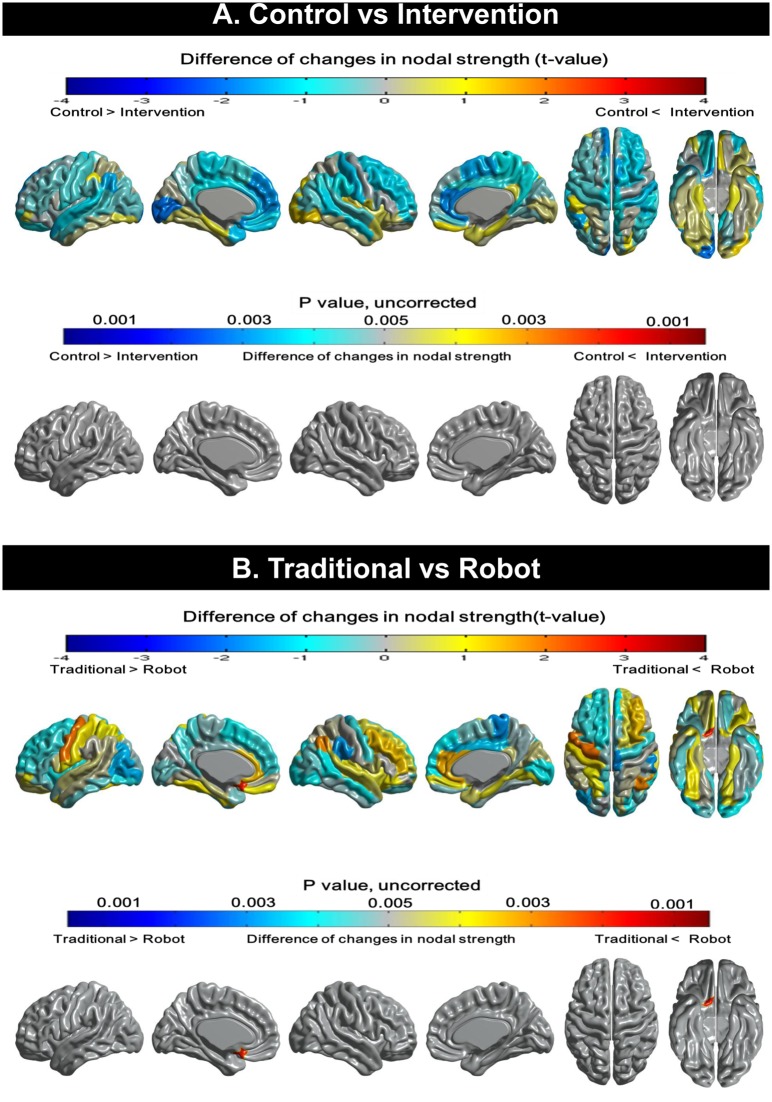
Topographical changes in nodal strength. (A) There are no significant differences in regional nodal strength between the control and the intervention group. (B)The robot group shows increased nodal strength in the rectus gyrus than the traditional group.

There were no significant changes in regional nodal strength in the intervention group compared to the control group (Fig 3A). Direct comparison of the traditional and robot groups indicated that the latter had greater nodal strength in the left rectus gyrus (Fig 3B).

### Effects of cognitive training on cognitive performance

The intervention group showed a greater improvement only in the executive function (SOC task) scores than the control group (*P* < 0.001) (Table 3). When the two intervention groups were directly compared to each other (Table 4), scores for the ADAS-cog (general cognitive scores) and PRM task (visual memory) revealed greater improvement in the traditional intervention group than in the robot group. In contrast, the robot group did not outperform the traditional group on any neuropsychological test.

**Table 3:** Changes in cognitive functions between the control and the intervention group.

	Control (n = 28)				Intervention group (n = 43)				*P* value for group	R^2
	Baseline	Post-Intervention	Delta (Post-Pre)	*P* value for delta	Baseline	Post-intervention	Delta (Post-Pre)	*P* value for delta		
**ADAS-Cog** ^**a**^	6.3 ± 3.6	4.6 ± 2.2	-1.6 ± 2.7	**0.002**	7.1 ± 3.6	5.2 ± 2.8	-1.9 ± 2.8	**< 0.001**	0.832	0.083
**Cambridge Neuropsychological Test Automated Battery**										
***Memory***										
**Delayed Matching to Sample (DMS)**										
** Correct response (%)**	72.9 ± 11.0	77.7 ±11.3	4.6 ± 12.9	0.054	72.5 ± 13.2	77.2 ± 14.1	4.8 ± 16.0	**0.044**	0.829	0.042
** DMS errors** ^**a**^	0.1 ± 0.2	0.1 ± 0.2	-0.0 ± 0.2	0.918	0.2 ± 0.2	0.1 ± 0.2	-0.0± 0.2	0.164	0.458	0.020
**Pattern Recognition Memory (PRM)**										
** Correct response (%)**	89.0 ± 7.6	91.1 ± 6.9	1.7 ±8.0	0.239	87.3 ± 15.0	92.5 ± 8.8	5.1± 14.7	**0.020**	0.279	0.098
**Paired Associate Learning (PAL)**										
** Total errors(adjusted**) ^**a**^	28.9 ± 25.3	25.6 ± 20.7	-0.7± 16.7	0.826	31.5 ±21.2	24.0 ± 17.0	-7.4 ± 18.2	**0.008**	0.053	0.125
***Attention***										
** Rapid Visual Information Processing (RVIP)**	0.9 ± 0.0	0.9 ± 0.0	0.0 ± 0.0	0.536	0.9 ± 0.1	0.9 ± 0.1	0.0 ± 0.1	0.110	0.124	0.052
** Reaction Time (RTI)** ^**a**^	360.1 ± 41.4	349.1 ± 48.9	-11.6 ± 49.3	0.192	344.7 ± 65.3	339.3 ± 59.9	-6.5 ± 55.4	0.427	0.385	0.051
***Executive Function***										
**Spatial Working Memory (SWM)**										
** Between errors** ^**a**^	46.8 ± 17.0	44.9 ± 14.5	-1.6 ± 15.9	**<0.001**	50.5 ± 21.6	45.8± 24.5	-4.7 ± 23.4	**<0.001**	0.750	0.134
** Strategy** ^**a**^	37.4 ± 3.1	37.0 ± 3.2	-0.2 ± 3.4	**<0.001**	38.2 ± 4.3	37.0 ± 3.5	-1.2 ± 4.7	**<0.001**	0.389	0.005
**Stocking of Cambridge (SOC)**										
** Problems solved**	7.7 ± 1.6	7.2 ± 1.7	-0.7 ± 1.5	**0.019**	6.5 ± 1.6	7.5 ± 1.6	1.0 ± 2.1	**0.004**	<0.001*	0.208

*P value < 0.05 adjusted by age, gender and education,

^a^Lower scores represent better performance.

**Table 4:** Changes in cognitive functions between the traditional and the robot group.

	Traditional (n = 23)				Robot (n = 20)				*P* value for group	R^2
	Baseline	Post-Intervention	Delta (Post-Pre)	*P* value for delta	Baseline	Post-intervention	Delta (Post-Pre)	*P* value for delta		
**ADAS-Cog** ^**a**^	7.5 ± 3.5	4.6± 2.6	-2.9 ± 2.3	**<0.001**	6.8 ± 3.8	5.7 ± 2.9	-0.9 ± 2.8	0.157	0.014*	0.234
**Cambridge Neuropsychological Test Automated Battery**										
***Memory***										
**Delayed Matching to Sample (DMS)**										
** Correct response (%)**	74.6 ± 10.0	76.4 ± 15.2	2.5 ± 15.6	0.440	71.1 ± 15.8	78.0 ± 13.1	7.3 ± 16.4	**0.045**	0.093	0.052
** DMS errors** ^**a**^	0.2 ± 0.2	0.1 ± 0.2	-0.0 ± 0.2	0.537	0.2 ± 0.2	0.1 ± 0.2	- 0.1 ± 0.2	0.149	0.337	0.057
**Pattern Recognition Memory (PRM)**										
** Correct response (%)**	85.4 ± 18.9	94.8 ± 5.8	9.4 ± 18.5	**0.021**	89.3 ± 9.3	90.0 ± 10.7	0.7± 7.2	0.633	0.017*	0.174
**Paired Associate Learning (PAL)**										
** Total errors(adjusted**) ^**a**^	28.7 ± 21.1	22.4 ± 12.2	-6.3 ± 18.7	0.115	34.4 ± 21.3	25.7 ± 18.3	-8.6 ± 18.0	**0.032**	0.942	0.109
***Attention***										
** Rapid Visual Information Processing (RVIP)**	0.9 ± 0.1	0.9 ± 0.0	0.0 ± 0.1	0.065	0.9 ± 0.1	0.9 ± 0.1	0.0 ± 0.0	**0.007**	0.904	0.177
** Reaction Time (RTI)** ^**a**^	344.0 ± 69.2	348.8 ± 74.2	-3.9 ± 55.8	0.732	335.0 ± 59.2	327.3 ± 38.5	-9.4± 56.2	0.441	0.807	0.065
***Executive Function***										
** Spatial Working Memory (SWM)**										
** Between errors** ^**a**^	52.0 ± 22.1	47.0 ± 21.4	-5.0 ± 24.0	**<0.001**	49.0 ± 21.4	44.6 ± 27.7	-4.4 ± 23.2	**<0.001**	0.906	0.093
** Strategy** ^**a**^	38.5 ± 4.4	36.7 ± 3.3	-1.8 ± 4.2	**<0.001**	37.9 ± 4.0	37.4 ± 3.8	-0.5 ± 5.1	**<0.001**	0.302	0.056
**Stocking of Cambridge (SOC)**										
** Problems solved**	6.7 ± 1.5	7.3 ± 1.6	0.5 ± 2.2	0.245	6.4 ± 1.7	7.7 ± 1.6	1.4 ± 2.0	**0.003**	0.230	0.080

* *P* value < 0.05 adjusted by age, gender and education,

^a^Lower scores represent better performance.

### Correlation of changes in cognitive performance with those in cortical thickness

**Fig 4:**
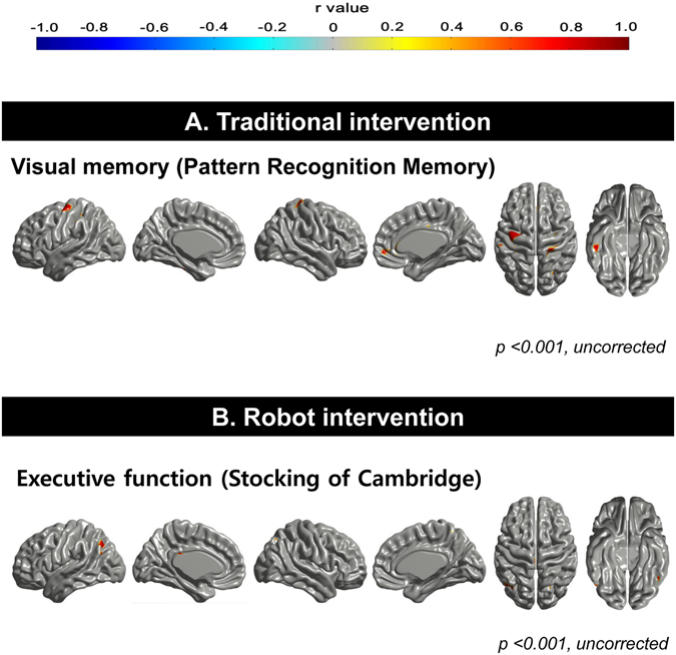
Correlation of changes in cognitive functions and changes in cortical thickness. (A) In the traditional group, changes in the raw scores of visual memory are positively correlated with those of cortical thickness in the right inferior temporalgyrus and right subgenual cingulate region (uncorrected *P* < 0.001). (B) For the robot group, changes in the raw scores of executive function are positively correlated with those of left temoporo-parietal junction as well as left inferior temporal gyrus (uncorrected P <0.001).

In the traditional group (Fig 4A), changes in the raw scores of visual memory (PRM task) were positively correlated with those of cortical thickness in the right inferior temporal gyrus and right subgenual cingulate region (uncorrected *P* < 0.001), whereas other changes in cognitive scores such as ADAS-cog and SWM task were not correlated with those in cortical thickness. For the robot group (Fig 4B), changes in the raw scores of executive function (SOC task) were positively correlated with those of left temoporo-parietal junction as well as left inferior temporal gyrus (uncorrected *P* < 0.001). Other scores were not correlated with changes in cortical thickness.

## Discussion

The first major finding of our study was that the groups of participants who received cognitive training showed less cortical thinning than the control group. More specifically, when the intervention group is compared to the control group, the intervention group showed less cortical thinning in heteromodal association cortices such as the right and left medial prefrontal and right middle temporal cortex (Fig 2). Heteromodal association cortices are responsible for integrating information from unimodal association cortex and paralimbic areas, which are important for facilitating learning [35]. The cognitive training programs used in this study may require strong integration of information as well as active interactions between neuronal networks because these interventions target multiple cognitive domains, which require activation and processing by the heteromodal association cortices. A previous study showed that lateral and medial prefrontal cortices as well as superior and middle temporal gyri were consistently affected during the aging process [36]. Therefore, reduced cortical thinning of the medial prefrontal cortex and middle temporal cortex in the intervention group suggests that multi-domain targeted cognitive training, such as those used in the current study, may counteract the age-associated structural changes in the elderly. The precise neurobiological mechanisms responsible for the changes of cortical thickness in older adults after cognitive training are not known. However, previous studies have suggested several explanations, such as changes in the size of neurons or glial cells as well as genesis of neurons or glial cells, or changes in vascularization [4, 5, 37, 38].

Our second major finding was that, although changes in the overall mean cortical thickness were not significantly different between the two groups that received cognitive interventions, the robot group, when compared to the traditional group, had attenuated cortical thinning bilaterally in the dorsal ACCs. There are several possible explanations for this difference. First, the robot-assisted intervention included more exercise-related programs than traditional training (Table 1). Physical exercise is known to promote cerebral blood flow especially in the ACC [39]. Therefore, the increased amount of physical activity in the robot group might have resulted in decreased cortical thinning in the ACC. Second, interaction with robots could have provided extra motivation for the elderly who had never been exposed to such advanced technology. According to prior studies, older individuals participating in cognitive intervention using a computerized or video game mentioned that these people were willing to learn new technology in the hope that they can empathize better with their grandchildren as well as improving their cognitive function [10]. It is well known that the ACC serves to motivate goal-directed behaviors [40]. Many patients with medial frontal lobe dysfunction are more impaired in self-initiated physical and cognitive activities than externally mediated activities (akinesia paradoxica) [41]. Hence, the greater motivation in the robot group may have activated the ACC more than the traditional training would have. Third, interacting with robots as well as using tablet computers could be sufficiently novel to induce additional brain activity in the elderly because using a computer requires multi-tasking abilities such as the allocation and shifting of attention [42]. Lesions studies of humans [43] and ablations studies in monkeys [44] as well as imaging studies in normal subjects [45] have revealed that the ACC plays an important role in the cortical network critical for allocating visual attention.

Another major finding was that the intervention group showed less reduction of global structural network topology, namely nodal strength, global efficiency, and clustering coefficient. Previous studies have shown that these characteristics of brain networks decrease significantly with normal aging [46, 47], which was replicated in our control group. Clustering coefficient is an index of segregation that is associated with local efficiency of information transfer and robustness; high global efficiency represents integration, which also suggests efficient conveyance of parallel information [21, 46]. The attenuated decline in nodal strength, global efficiency and clustering coefficient in our intervention groups suggests that the cognitive training might delay age-related alterations of brain networks.

We also found that the nodal strength in the left rectus gyrus was increased in the robot group compared to the traditional group. The ventromedial orbitofrontal cortex including the rectus gyrus has previously been shown to contribute to reward-based decision-making and motivation [48–51]. It is possible that more frequent individual feedback including announcing the winner of the month in the robot group could have activated the reward system more than the traditional group. It is also plausible that engaging in new technology such as the robotic system or computers may provide greater motivation for the elderly who have not been exposed to those novel environments, which could result in strengthened neural connectivity in the rectus gyrus, contributing to motivation control of goal-directed behavior [48, 51]. It is also noteworthy that the rectus gyrus is an extension of the ACC to the frontal lobe [52], which may help explain the simultaneous changes in both regions that we observed.

When the intervention group was compared to the control group, the intervention group showed greater improvement in the executive function (SOC task). The SOC subtest was designed to measure the planning efficiency and problem solving ability [53]. Therefore, the improvement of SOC task in the intervention group would suggest that multi-domain cognitive training might help improve executive functions in the elderly. These results could be compatible with the previous results showing that multi-domain cognitive training improves executive functions such as reasoning and processing speed [7, 54]. Direct comparison of the traditional and the robot groups indicated larger improvement in visual memory and general cognitive function in the traditional group. The reasons are unknown and further study is required to elucidate such differences.

Finally, changes of visual memory scores in the traditional group were positively correlated with changes in cortical thickness of the right inferior temporal and the right subgenual ventromedial prefrontal cortex, while improvements of the executive function in the robot group were significantly correlated with cortical thickening in the left temporo-parietal junction and right inferior temporal gyrus. Correlation does not directly imply causation, but perhaps the correlation in the traditional group can be explained by the fact that the inferior temporal cortex is involved in visual shape categorization [55] while the subgenual ventromedial prefrontal cortex plays a role in memory consolidation [56, 57]. Interestingly, all of the correlated areas that we observed were located in the right hemisphere which predominantly processes visual information [58]. On the other hand, the improvement of executive functions in the robot group may be related to the changes in the left temporo-parietal junction that mediates the allocation of attention to task-relevant information [59], which is critical for planning and problem solving [60].

This study has several limitations. First, it was not possible to control participants’ daily cognitive activity at home such as using computers or reading books. Second, although there was no significant difference in proportions of gender between the control and two intervention groups, the larger portion of women in the control group might have influenced our results since previous studies have revealed that cortical thinning occurs faster in women than in men [61]. However, we adjusted the gender as a covariate to minimize gender effects on cortical thinning.

In conclusion, although several studies have revealed that social robots improve the mood of the elderly [62], prior to this study, there have been no studies that have investigated the effects of robot-assisted cognitive training on structural brain changes in the elderly. The results of this study have revealed that cognitive training can mitigate cortical thinning and network alterations in the elderly, providing evidence that training-driven plasticity can occur in the elderly. Furthermore, our results suggest that robot-assisted cognitive training can help alleviate cortical thinning in the ACC compared to the traditional one, which is important in initiation and persistence of goal-directed activities as well as the allocation of attention. Although further research is needed to improve these cognitive training robots, with the dramatic increase in the number of older people, robot-assisted cognitive training, as well as the traditional one, may help reduce the cognitive disabilities associated with aging.

## Supporting Information

S1 CONSORT Checklist CONSORT Checklist. (DOC)

S1 Fig The two robots used for cognitive training. (TIF)

S1 Methods Detailed descriptions about traditional and robot-assisted interventions. (DOC)

S2 Methods Detailed descriptions of Cambridge Neuropsychological Test Automated Battery (CANTAB). (DOC)

S3 Methods Detailed procedures of network analyses. (DOC)

S1 Protocol Trial Protocol (Summary in English). (DOC)

S1 Table Changes in global structural connectivity using a graph theoretical approach. (DOC)
