# Supplementary figures and images for: Structural Brain Changes after Traditional and Robot-Assisted Multi-Domain Cognitive Training in Community-Dwelling Healthy Elderly
Source: PLoS One. 2015 Apr 21;10(4):e0123251. doi: 10.1371/journal.pone.0123251 (PMC4405358; doi:10.1371/journal.pone.0123251)

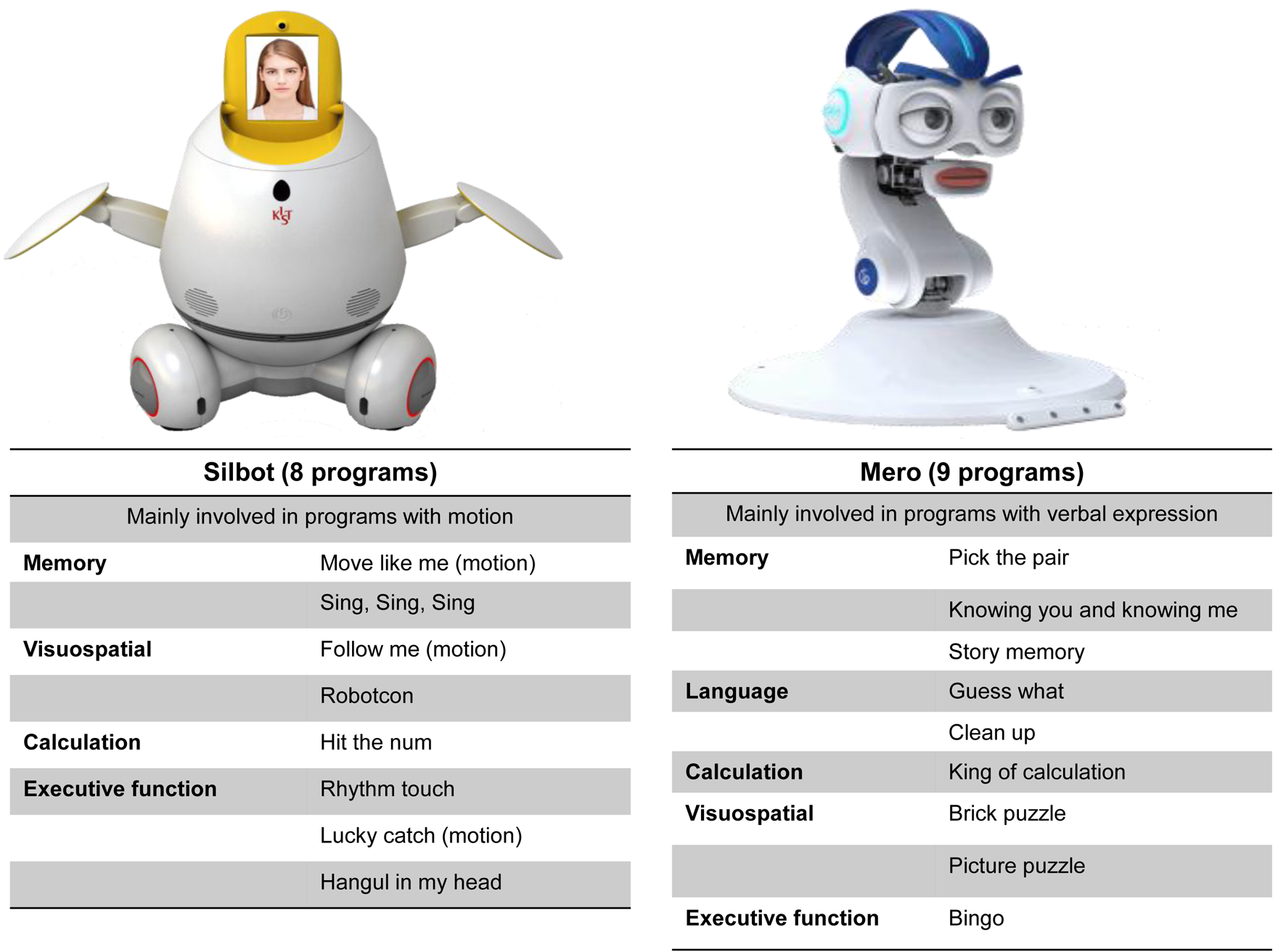

Supplement: S1 Fig — (TIF) [file pone.0123251.s002.tif]
