# Supplementary material for: Structural Brain Changes after Traditional and Robot-Assisted Multi-Domain Cognitive Training in Community-Dwelling Healthy Elderly
Source: PLoS One. 2015 Apr 21;10(4):e0123251. doi: 10.1371/journal.pone.0123251 (PMC4405358; doi:10.1371/journal.pone.0123251)
Supplement: S1 Methods — (DOC) [file pone.0123251.s003.doc]

**S1 METHODS**

**1. INTERVENTIONS**

**1) Traditional Interventions**

In traditional cognitive training, the psychometricians displayed questions on a screen that was set-up in the front of the intervention room, and then participants answered the questions either verbally or in written form. The psychometricians then presented the answers on the screen.

Memory training in this group comprised of verbal episodic memory, visual memory, logical memory (story memory), and paired associate learning. For example, participants were taught mnemonic strategies for remembering word lists or details of stories, identifying the places of objects in a previously seen picture, and matching other participants’ faces with their own characteristics such as name, age, or hometown. Language function training focused on the ability to generate words in given phonemic or semantic categories. For instance, tasks included crossword puzzles, guessing the right word after listening to several explanations for the word, and making up creative stories with given words. Reasoning training focused on the ability to solve problems that followed a serial sequence of patterns or letters. For example, the participants were required to decode symbols and then translate to Korean letters. Working memory training involved performing tasks that required monitoring or manipulation of verbal information such as summarizing long stories including the main ideas, performing mental arithmetic with numbers of several digits, and so on. Speed of processing training was represented by the ability to identify and manage visual or auditory information quickly and correctly. For example, participants were asked to clap every time they heard several determined words in the form of long stories or to identify one different item among similar items on a screen as fast as they could. Calculation training focused on addition, subtraction, multiplication, and division, which was practiced by calculating the total money expenditures as if they were shopping in a department store. Visuospatial function training was related to spatial representation or visuo-constructions, such as drawing maps from their houses to the nearest subway station, and mental rotation of objects.

**2) Robot Interventions**

A total of 17 cognitive training programs were used in the robot-assisted cognitive training, which consisted of five programs for memory, two for language, two for calculation, four for visuospatial function and four for executive function.

The memory training programs were composed of the following five programs: “Move like me”, in which the participants had to memorize the sequential movement of the robot and then copy its sequence as much as they could; “Pick the pair”, which required participants to remember pairs of words and pick matching words displayed on the screen; “Knowing you and knowing me”, which directed participants to learn and memorize the personal information of other participants including their name, home town, favorites etc.; “Story memory” that required participants to remember specific stories related to episodes or general public knowledge such as historical famous persons, medical information, and so on, and finally “Sing, Sing, Sing” in which participants had to fill in the blanks in lyrics after the robot played a song.

Language training was conducted mainly using the two programs of “Guess what”, which involved guessing a target word after listening to ten hints related to the word, and “Clean up”, which was designed to develop listening comprehension and working memory by performing complex instructions that the robot verbalized.

Calculation training programs were performed using the “King of calculation”, which required participants to mentally add several numbers that the robot presented verbally, while “Hit the Num”, a modified version of the N-back task, required the subject to calculate the current digit and the one from *n* steps earlier in response to a sequenced auditory stimulus of numbers.

The four visuospatial function training programs that were implemented were as follows. In “Follow me”, participants had to remember the route taken by the robot and duplicate the route from the start to the end points; “Robotcon” required the participants to memorize the route of the robot and move the robot in the memorized route using control buttons on their smart pad; “Brick puzzle” instructed patients to fill out empty squares with puzzle bricks; and “Picture puzzle” instructed patients to match puzzle pieces, and complete a jigsaw puzzle.


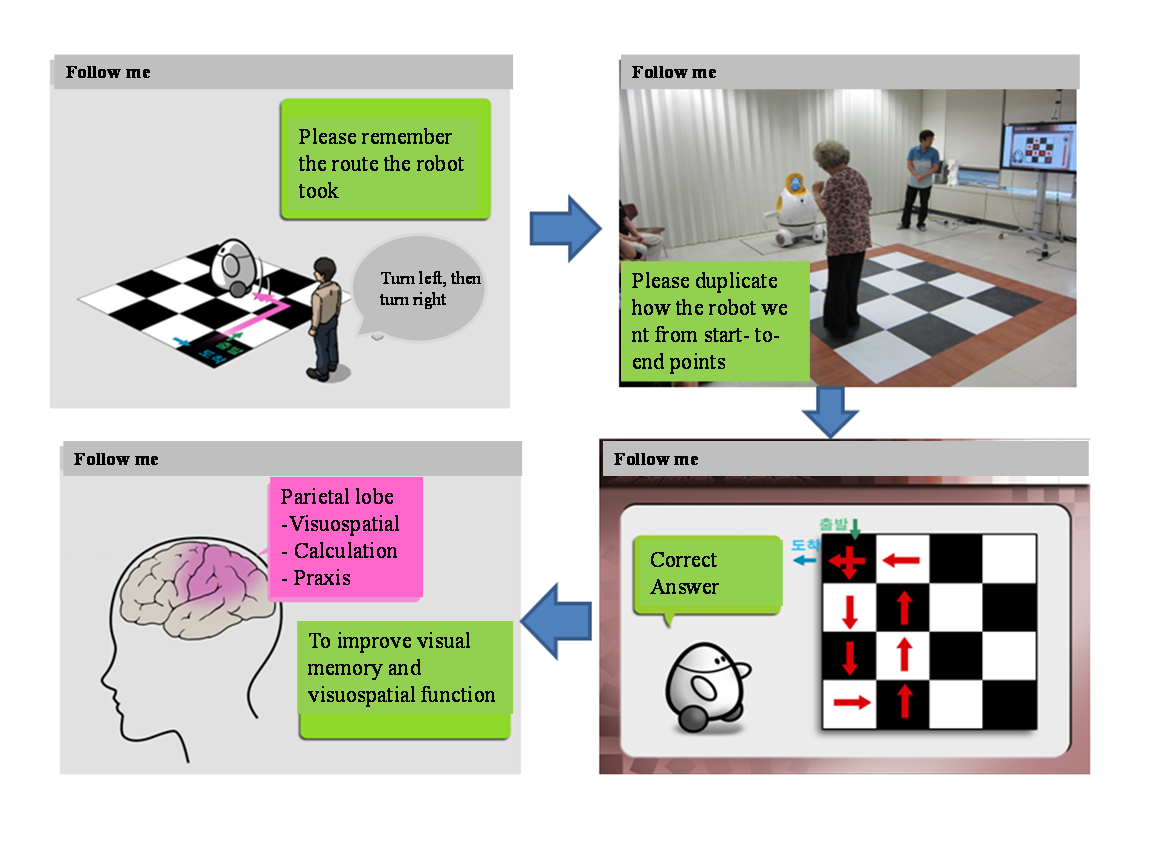


The following four programs were used in the training of executive functions. Working memory was trained with the programs “Rhythm Touch”, in which participants were instructed to hit numbers on the smart pad matching the pattern of serial numbers verbalized by the robot. Speed of processing was a component of all programs, especially for “Lucky catch”, which required participants to touch lucky bags on the screen as many times as possible within a specified amount of time. “Hangul in my head” required participants to calculate the number of strokes in the Korean letter the robot presented (then the letter disappeared within a few seconds), which requires working memory function as well as calculation and language. The game “Bingo” was modified to develop reasoning training by asking the participants to look at the other participants’ BINGO boards on a screen and choose the number to achieve the BINGO.

Each program was divided into various levels of difficulty, so the difficulty was raised each time a participant achieved a certain criterion of performance on a particular task. Most of these programs are based on training activities that have been previously used at Samsung Medical Center. Two neurologists, four clinical neuropsychologists, and two language pathologists reached consensus in determining detailed contents of each robotic program.
