# Supplementary material for: Structural Brain Changes after Traditional and Robot-Assisted Multi-Domain Cognitive Training in Community-Dwelling Healthy Elderly
Source: PLoS One. 2015 Apr 21;10(4):e0123251. doi: 10.1371/journal.pone.0123251 (PMC4405358; doi:10.1371/journal.pone.0123251)
Supplement: S2 Methods — (DOC) [file pone.0123251.s004.doc]

**S2 Methods. NEUROPSYCHOLOGICAL TESTS**

**Cambridge Neuropsychological Test Automated Battery (CANTAB)**

We used seven tests from the CANTAB, which included the three visual memory tasks of Delayed Matching to Sample (DMS), Pattern Recognition Memory (PRM), and Paired Associates Learning (PAL), two executive function or working memory tests, namely Spatial Working Memory (SWM) and Stockings of Cambridge (SOC), and two attention tests, namely Reaction Time (RTI) and Rapid Visual Information Processing (RVIP). Detailed descriptions of the tests are available on Cambridge Cognition's website (<http://www.cambridgecognition.com/academic/cantabsuite/tests>).

**① Delayed matching to sample (DMS).** The subject is asked to touch the pattern that matches the sample. In some trials the sample and the choice patterns are shown simultaneously, whereas in others a delay (of 0, 4, or 12 seconds) is introduced between sample and the choice patterns. This task measured visual memory function in a four-choice delayed recognition memory paradigm. *The percentage of correct solutions* for all delay conditions gives a good overall impression of visual memory ability. The higher the score, the better the test results. *DMS Errors* reported the number of errors. An error occurred if the subject did not select the correct box in the first response. A lower score is better.

**② Rapid Visual Information Processing (RVP).** This is a visual continuous performance task. Subjects are instructed to detect target sequences of digits (for example, 2-4-6, 3-5-7, 4-6-8) and to register responses using the press pad. Target sequences occur at the rate of 16 every 2 minutes. *A′ (A prime)* is the signal detection measure of sensitivity to the target, regardless of response tendency (range 0.00 to 1.00; bad to good). In essence, this measure is a measure of how efficient the subject is at detecting target sequences.

**③ Pattern recognition memory (PRM).** This tests visual recognition memory in a two-choice forced discrimination paradigm. A white box appears in the center of the computer screen, inside which digits, from 2 to 9, appear in a pseudo-random order, at the rate of 100 digits per minute. The test is in two parts; a ‘warm-up’ practice phase which lasts for two minutes (slow mode, five minutes) and is not scored, and a test phase which lasts for four, six or ten minutes, depending on the test mode, the last three/five/seven and a half of which are assessed. The subject is presented with a series of 12 visual patterns, one at a time, in the center of the screen. These patterns are designed so that they cannot easily be given verbal labels. In the recognition phase, the subject is required to choose between a pattern they have already seen and a novel pattern. In this phase, the test patterns are presented in the reverse order of the original order of presentation. *PRM Percent correct* measures the number of correct responses, expressed as a percentage, with higher values indicating better pattern of recognition memory.

**④ Reaction time (RTI).** In this test, the subject's speed of response to a visual target where the stimulus is either predictable (simple reaction time) or unpredictable (choice reaction time) is evaluated. *Five-choice reaction time* is the speed with which the subject releases the press pad button in response to a stimulus in any one of five locations. A lower time is better.

**⑤ Spatial working memory (SWM).** This task assesses the subject's ability to retain spatial information and to manipulate remembered items in working memory. The subject must touch each box in turn until one opens with a blue token inside (a search). When a blue token has been found, the subject has to place it in the right column (‘home’) by touching the right-hand side of the screen. The subject must then begin a new search for the next blue token. It may be in any of the boxes that so far have been empty. This is repeated, until a blue token has been found in every box on the current screen. Touching any box in which a blue token has already been found is an error. The subject decides the order in which the boxes are searched. The computer determines the number of empty boxes that must be visited (discounting errors). Performance at the harder levels of this task is enhanced by the use of a heuristic search strategy. *SWM Between errors* are defined as times the subject revisits a box in which a token has previously been found. Lower is better. *SWM Strategy* is obtained by counting the number of times the subject begins a new search with a different box for six- and eight-box problems only. A high score represents poor use of this strategy and a low score equates to effective use of strategy.

**⑥ Stockings of Cambridge (SOC).** The task is similar to the ‘Tower of London’ test and assesses the subject's ability to engage in spatial problem solving. The subject must use the balls in the lower display to copy the pattern shown in the upper display. The balls may be moved one at a time by touching the required ball, then touching the position to which it should be moved. The time taken to complete the pattern and the number of moves required are taken as measures of the subject’s planning ability. This test makes substantial demands on executive function. ‘*Problems solved in a minimum number of moves’* is the fundamental measure, and records the number of occasions upon which the subject successfully completes a test problem in the minimum possible number of moves. A higher score is better.

**⑦ Paired associate learning (PAL).** This test assesses simple visual pattern and visuospatial associative learning, which contains aspects of both delayed response procedure and a conditional learning task. For each stage, boxes are displayed on the screen. All are opened in a randomized order. One or more of them will contain a pattern. The patterns shown in the boxes are then displayed in the middle of the screen, one at a time, and the subject must touch the box where the pattern was originally located. Each stage may have up to 10 attempts (trials) in total (the first presentation of all the shapes, then up to 9 repeat presentations). If the subject makes an error, the patterns are re-presented to remind the subject of their locations. When the subject gets all the locations correct, they proceed to the next stage. If the subject cannot complete a stage correctly, the test terminates. *PAL Total errors* reports the total number of errors. Lower is better.
