# Supplementary material for: Structural Brain Changes after Traditional and Robot-Assisted Multi-Domain Cognitive Training in Community-Dwelling Healthy Elderly
Source: PLoS One. 2015 Apr 21;10(4):e0123251. doi: 10.1371/journal.pone.0123251 (PMC4405358; doi:10.1371/journal.pone.0123251)
Supplement: S3 Methods — (DOC) [file pone.0123251.s005.doc]

**S3 Methods. GRAPH ANALYSIS**

**1) Structural connectivity network construction**

① *Network node definition.*

We used the automated anatomical labelling (AAL) template [1] to parcellate the whole cerebral cortex into 78 areas (39 regions in each hemisphere). Individual T1-weighted images were nonlinearly registered to the ICBM152 T1 template in the MNI space. The AAL atlas was transformed from the MNI space to the T1 native space using inverse transformation with a nearest-neighbour interpolation method.

② *Network edge definition*

Distortions in the diffusion tensor images caused by eddy currents and simple head motions were corrected by the diffusion toolbox of the FSL package (www.fmrib.ox.ac.uk/fsl/fdt). Diffusion tensor models were estimated and the fractional anisotrophy(FA) and the apparent diffuse coefficient (ADC) were calculated at each voxel. We reconstructed whole-brain white matter fibre tracts in native diffusion space for each subject using the fibre assignment by continuous tracking algorithm [2], which is embedded in the Diffusion Toolkit (trackvis.org) [3]. We terminated tracking when the angle between two consecutive orientation vectors was greater than the given threshold of 45° or when both ends of the fibres extended outside of the white matter mask that was generated by a tissue segmentation process.

③ *Network construction*

T1-weighted images were coregistered to b0 images in the DTI space using linear registration. Reconstructed whole-brain fibre tracts were inversely transformed into the T1 space and the fibre tracts and AAL-based parcellated regions were located in the same space. Two nodes (regions) were considered to be structurally connected by an edge when at least the end points of three fibre tracts were located in these two regions. A threshold of the number of fibre tracts was selected to reduce the risk of false-positive connections due to noise or the limitations of deterministic tractography [4,5]. The number of fibre tracts and FA were calculated for a weight of each edge between two nodes. Fibre number determined by streamline tracking may reflect the WM structure [6], and has been used previously as a weight for network edges [4,7-9]. The FA value is an important index to evaluate fibre integrity [10,11] and has a high correlation with conductivity [12]. In this study, the value obtained by multiplying the fibre number by the mean FA along all the fibres connecting a pair of regions was used to weight the edge. Finally, weighted structural networks represented by symmetric 78 x 78 matrices were constructed for each individual.

**2) Network analysis**

*① Nodal strength*

We first computed the most fundamental and basic network measure, nodal strength. Nodal strength is defined as the sum of all neighbouring edge weights of a node and is a measure of the local quantity of a network. The global mean strength of the network was also measured by the average of the local strength of each node within the network.

*② Global efficiency*

We calculated global efficiency as a measure of network integration. We measured shortest path lengths between all pairs of nodes in the network and computed the global efficiency as the average inverse shortest path length [13].

*③ Clustering coefficient*

To measure network segregation, the weighted clustering coefficient of a node, which was defined as the likelihood of the neighbourhoods being connected to each other or not [14], was computed. We measured the mean clustering coefficient of a network as the average of the clustering coefficients across all nodes.

Because we investigated longitudinal changes in the network for each individual, we adopted the following strategy. We hypothesized that the number of edges and network density did not change within the 3 months. It is biologically and anatomically plausible that within this timeframe, there is no physical generation or disappearance of axonal fibres, but only changes in the efficacy of structural connections in old brains are detected [15]. We adopted edges detected in both the baseline and the post-intervention networks, but otherwisethe weights of edges were set to 0 (AND operation). Hence, baseline and post-intervention structural connection matrices had an equal number of edges, but with variations in connection efficacy.

**REFERENCES**

1. Tzourio-Mazoyer N, Landeau B, Papathanassiou D, Crivello F, Etard O, et al. (2002) Automated anatomical labeling of activations in SPM using a macroscopic anatomical parcellation of the MNI MRI single-subject brain. Neuroimage 15: 273-289.

2. Mori S, Crain BJ, Chacko VP, van Zijl PC (1999) Three-dimensional tracking of axonal projections in the brain by magnetic resonance imaging. Ann Neurol 45: 265-269.

3. Wang R, Beener T, Sorensen AG, Wedeen VJ (2007) Diffusion toolkit: a software package for diffusion imaging data processing and tractography. Proc Intl Soc Mag Reson Med 15: 3720.

4. Shu N, Liu Y, Li K, Duan Y, Wang J, et al. (2011) Diffusion tensor tractography reveals disrupted topological efficiency in white matter structural networks in multiple sclerosis. Cereb Cortex 21: 2565-2577.

5. Lo CY, Wang PN, Chou KH, Wang J, He Y, et al. (2010) Diffusion tensor tractography reveals abnormal topological organization in structural cortical networks in Alzheimer's disease. J Neurosci 30: 16876-16885.

6. Houenou J, Wessa M, Douaud G, Leboyer M, Chanraud S, et al. (2007) Increased white matter connectivity in euthymic bipolar patients: diffusion tensor tractography between the subgenual cingulate and the amygdalo-hippocampal complex. Mol Psychiatry 12: 1001-1010.

7. Batalle D, Eixarch E, Figueras F, Munoz-Moreno E, Bargallo N, et al. (2012) Altered small-world topology of structural brain networks in infants with intrauterine growth restriction and its association with later neurodevelopmental outcome. Neuroimage 60: 1352-1366.

8. Yan C, Gong G, Wang J, Wang D, Liu D, et al. (2011) Sex- and brain size-related small-world structural cortical networks in young adults: a DTI tractography study. Cereb Cortex 21: 449-458.

9. Zhang Z, Liao W, Chen H, Mantini D, Ding JR, et al. (2011) Altered functional-structural coupling of large-scale brain networks in idiopathic generalized epilepsy. Brain 134: 2912-2928.

10. Basser PJ, Pierpaoli C (1996) Microstructural and physiological features of tissues elucidated by quantitative-diffusion-tensor MRI. J Magn Reson B 111: 209-219.

11. Beaulieu C (2002) The basis of anisotropic water diffusion in the nervous system - a technical review. NMR Biomed 15: 435-455.

12. Tuch DS, Wedeen VJ, Dale AM, George JS, Belliveau JW (2001) Conductivity tensor mapping of the human brain using diffusion tensor MRI. Proc Natl Acad Sci U S A 98: 11697-11701.

13. Latora V, Marchiori M (2001) Efficient behavior of small-world networks. Phys Rev Lett 87: 198701.

14. Onnela JP, Saramaki J, Kertesz J, Kaski K (2005) Intensity and coherence of motifs in weighted complex networks. Phys Rev E Stat Nonlin Soft Matter Phys 71: 065103.

15. Hagmann P, Grant PE, Fair DA (2012) MR connectomics: a conceptual framework for studying the developing brain. Front Syst Neurosci 6: 43.
