# Supplementary material for: Structural Brain Changes after Traditional and Robot-Assisted Multi-Domain Cognitive Training in Community-Dwelling Healthy Elderly
Source: PLoS One. 2015 Apr 21;10(4):e0123251. doi: 10.1371/journal.pone.0123251 (PMC4405358; doi:10.1371/journal.pone.0123251)

## PROTOCOL SUMMARY

.

| **Title:** | **Structural brain changes after a robot-assisted cognitive training in the elderly(ROSE study)** |
| --- | --- |
| **Précis:** | Cognitive training in the elderly has been shown to improve cognitive functioning. Most previous studies demonstrated this improvement using cognitive tests; few studies have employed brain structural changes of magnetic resonance images as outcome measures. With the advent of robotics, service robots that can interact with humans have attracted both industry and academic interest. In particular, robots to assist the elderly may be important considering the rapid increase in the aging population and the exorbitant healthcare costs associated with caring for older individuals with cognitive decline. For this reason, we have developed a total of 17 robot-assisted cognitive training programs for the elderly. |
| **Objectives:** | The goal of this study was to test the hypothesis that multi-domain cognitive training would delay age-associated cortical thinning and structural network alterations in the brains of the elderly. In addition, we also wanted to investigate if robot-assisted cognitive training would result in greater effects than traditional cognitive training.  **Primary Outcome Measures:**  Primary outcome: Primary outcomes were changes in cortical thickness   - [ Time Frame: between the baseline and the post-intervention assessment (12 week training) ] - [ Designated as safety issue: No ]   **Secondary: Structural network topology and Cognitive functions**  Further secondary outcomes were changes in the structural brain network and cognitive functions. Network topology was assessed by a graph theoretical approach between the baseline and the post-intervention assessment. Cognitive functions were measured by validated neuropsychological tests such as Cambridge Neuropsychological Test Automated Battery (CANTAB). |
| **Population:** | **Sample size calculation**  We calculated our sample size based on a comparison of changes in cortical thickness from baseline to follow-up between the control and the intervention groups, as well as within the training groups comparing the traditional and the robot groups. Assuming the standard deviation (SD) of 0.004 mm for changes in cortical thickness from our previous study , and we expected changes in cortical thinning of -0.005 mm (0.6 months required for change of -0.001mm) for the control group, -0.004 mm (0.74 months required for change of -0.001 mm) for the traditional intervention group, and -0.001 mm (3 months required for change of -0.001 mm) for the robot-assisted intervention group. Therefore, the planned enrolment for the study was 24 per intervention group, and 37 for control group to account for 10 % drop-out rate, which had 80 % power under the 5 % significance level.  Eligibility   | Ages Eligible for Study: | 60 Years and older | | --- | --- | | Genders Eligible for Study: | Both | | Accepts Healthy Volunteers: | Yes |   **Inclusion Criteria:**   - 1) community-dwelling volunteers aged 60 years or older without dementia - 2) MMSE=26 or above - 3) more than 6 years of education   **Exclusion Criteria:**   - 1) if they had known dementia or significant cognitive impairment accompanied by dysfunction of daily living activities; - 2) illiterate - 3) unavailable during the testing and intervention periods of the study; - 4) had severe losses in vision or hearing - 5) had major neurological or psychiatric illness history including any history of stroke, transient ischemic attack or traumatic brain injury; - 6) had a medication history which might affect on cognitive function such as acetylcholinesterase inhibitor or memantine - 7) had medical problems such as thyroid, liver and renal disease; - 8) had a significant structural abnormalities on their baseline brain MRI. |
| **Number of Sites:** | Single site (Gangnam Center for Dementia , one of public facilities for dementia prevention in Seoul, which is supported by Samsung Medical Center) |
| **Study Duration:** | From March 2011 to May 2012 |
| **Subject Participation Duration:** | Baseline evaluation : within 2 weeks before intervention  Intervention : 12 weeks  Post-intervention assessment : within 2 weeks after termination of intervention |
| **Description of Agent or Intervention:** | | [**Arms**](http://www.clinicaltrials.gov/ct2/help/arm_group_desc) | [**Assigned Interventions**](http://www.clinicaltrials.gov/ct2/help/interventions_desc) | | --- | --- | | Experimental: 24 persons with robot- intervention | Behavioral: Robot assisted cognitive training | | Active Comparator: 24 persons with conventional intervention  : conventional cognitive training group | Behavioral: Conventional cognitive training group | | 37 without intervention: Control group |  |   . |
| **Estimated Time to Complete Enrollment:** | 7 months |

***Schematic of Study Design:**


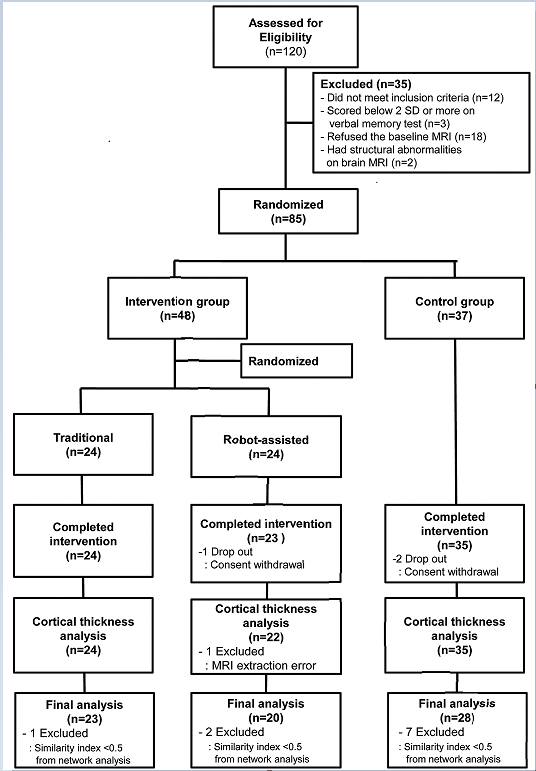

Supplement: S1 Protocol — (DOC) [file pone.0123251.s006.doc]
