# Supplementary material for: Structural Brain Changes after Traditional and Robot-Assisted Multi-Domain Cognitive Training in Community-Dwelling Healthy Elderly
Source: PLoS One. 2015 Apr 21;10(4):e0123251. doi: 10.1371/journal.pone.0123251 (PMC4405358; doi:10.1371/journal.pone.0123251)
Supplement: S1 Table — (DOC) [file pone.0123251.s007.doc]

**S1 Table.** Changes in global structural connectivity using a graph theoretical approach.

|  |  | Delta, Intervention group (n = 43) | | | | Delta, Control (n = 28) | *P* value for group  (Control vs Intervention) |
| --- | --- | --- | --- | --- | --- | --- | --- |
|  |  | Total  (n = 43) | Traditional  (n = 23) | Robot  (n = 20) | *P*-value  (Traditional  vs Robot) |
| **Nodal Strength** | | -6.7± 19.4 | -6.6 ± 16.7 | -6.9 ± 22.4 | 0.963 | -15.9± 17.2 | 0.030* |
| **Global Efficiency** | | -0.4 ± 1.2 | -0.3 ± 1.1 | -0.4 ± 1.4 | 0.096 | -0.9 ± 1.1 | 0.042* |
| **Clustering coefficient** | | -0.3± 1.1 | -0.3 ± 1.2 | -0.2 ± 1.1 | 0.678 | -0.9 ± 1.1 | 0.023* |

*P value < 0.05 adjusted by age and gender
